# Effect of Arsenic Soil Contamination on Stress Response Metabolites, 5-Methylcytosine Level and CDC25 Expression in Spinach

**DOI:** 10.3390/toxics11070568

**Published:** 2023-06-29

**Authors:** Marek Popov, Jan Kubeš, Pavla Vachová, František Hnilička, Veronika Zemanová, Jana Česká, Lukáš Praus, Marie Lhotská, Jiří Kudrna, Barbora Tunklová, Karel Štengl, Jiří Krucký, Tomáš Turnovec

**Affiliations:** 1 Department of Botany and Plant Physiology, Faculty of Agrobiology, Food and Natural Resources, Czech University of Life Sciences Prague, Kamýcká 129, 165 00 Praha-Suchdol, Czech Republic; 2 Department of Agroenvironmental Chemistry and Plant Nutrition, Faculty of Agrobiology, Food and Natural Resources, Czech University of Life Sciences Prague, Kamýcká 129, 165 00 Praha-Suchdol, Czech Republic; 3 Laboratory of Environmental Chemistry, Faculty of Agrobiology, Food and Natural Resources, Czech University of Life Sciences Prague, Kamýcká 129, 165 00 Praha-Suchdol, Czech Republic

**Keywords:** secondary metabolites, membrane damage, epigenetics, arsenic species, spinach, CDC25

## Abstract

Experimental spinach plants grown in soil with (5, 10 and 20 ppm) arsenic (As) contamination were sampled in 21 days after As(V) contamination. Levels of As in spinach samples (from 0.31 ± 0.06 µg g^−1^ to 302.69 ± 11.83 µg g^−1^) were higher in roots and lower in leaves, which indicates a low ability of spinach to translocate As into leaves. Species of arsenic, As(III) and As(V), were represented in favor of the As (III) specie in contaminated variants, suggesting enzymatic arsenate reduction. In relation to predominant As accumulation in roots, changes in malondialdehyde levels were observed mainly in roots, where they decreased significantly with growing As contamination (from 11.97 ± 0.54 µg g^−1^ in control to 2.35 ± 0.43 µg g^−1^ in 20 ppm As). Higher values in roots than in leaves were observed in the case of 5-methylcytosine (5-mC). Despite that, a change in 5-mC by As contamination was further deepened in leaves (from 0.20 to 14.10%). In roots of spinach, expression of the CDC25 gene increased by the highest As contamination compared to the control. In the case of total phenolic content, total flavonoid content, total phenolic acids content and total antioxidant capacity were higher levels in leaves in all values, unlike the roots.

## 1. Introduction

Arsenic (As) belongs to a group of metalloid elements, depending on the surrounding environment mostly present in arsenite As(III) and arsenate As(V) forms. Some of these compounds found application in agriculture for their toxic properties as pesticides or herbicides and medicine as antibiotics and antiparasitic drugs [1,2]. Naturally, As compounds are present in bedrock, whose erosion releases these forms into the environment, but anthropogenic sources have the potential to increase the levels of As significantly. Toxic metals can enter the human food chain by consuming plants grown in contaminated areas and be harmful to human health. Spinach is one of the plants where the presence of As was found in plants from affected regions [1,2,3,4,5].

The absorption of As compounds into plants is also dependent on their form when arsenate ones are able to use transporters for phosphate and molecules with arsenites (As(OH_3_)) passing through aquaglyceroporin channels [6,7]. The toxic effects of As have some different targets, such as the production of energy in the form of ATP through the disruption of oxidative phosphorylation, binding to -SH groups of proteins and the increase of oxidation stress through the creation of free radicals such as reactive oxygen species (ROS). These particles are created through physiological processes; however, in conditions of their higher production or decreased degradation, they can affect various biomolecules and impair their structure and function. Arsenic compounds in a plant cell can participate in ROS creation through the reduction of As(V) to As(III) [8,9,10,11] and the involvement of cytochrome oxidase and oxygen in mitochondria and chloroplast or methylation of As compounds, where these molecules react with O_2_ and produce ROS too [10,12].

One of the possible targets of ROS is cell membranes surrounding cells themselves as well as specific organelles with single (vacuole) or outer and inner membranes (mitochondria, chloroplast) [8,13]. The double bond between carbons of membrane lipids’ unsaturated fatty acids is the site where ROS such as superoxide radical, hydrogen radical or the other ones attack. Products of this cleavage could be alkoxy radicals, participating in a chain reaction, and other molecules such as malondialdehyde (MDA), which also serves as a marker of cell membrane damage by the effect of stress from various causes [13,14,15]. Plants and also other organisms developed several strategies for protecting the structure and functionality of cells and their parts. Besides the enzymes such as catalase, superoxide dismutase or guaiacol peroxidase, plants synthesize non-enzymatic compounds that are able to participate in the protection of biomolecules against ROS or they affect the source of free radicals more directly, for example, by the chelating of metals [8,16].

Different products of primary and secondary metabolism could be included here, such as tripeptide glutathione (γ-glutamylcysteinylglycine; GSH), terpenoid pigments such as carotenoids, ascorbic acid belonging to the group of vitamins, and a wide group of polyphenols, compounds originated from shikimate pathway, including phenolic acids, flavonoids, coumarins and others [8]. Thanks to their structure, some of the phenolics are able to stabilize free radicals and create chelates through bonds between hydroxyl groups and metals, which participate in ROS production [17]. The presence of compounds such as caffeic and ferulic acid or hyperoside (quercetin-3-galactoside) and rutin (quercetin-3-rutinoside) can participate in the antioxidant properties of spinach [18] and protect plants against various stress factors.

Epigenetic changes in DNA methylation levels have an important role in the adaptation of plants to stress. Methyl group addition or removal, mostly in the fifth carbon position, is mediated by the methyl group adding the DNA methyltransferase family of enzymes and the methyl group removing ten–eleven translocation (TET) and the thymine–DNA glycosylase (TDG) families of enzymes. Their activity can differ by abiotic stress source, such as the presence of toxic elements [19,20,21,22,23,24]. The DNA methylation level can affect gene expression, which is increasing with DNA demethylation or lowering in the case of DNA hypermethylation leading to gene silencing. Changes in methylation status can be manifested by suppression or stimulation of the growth of plants or their parts and can affect phenological stages and plants’ reproducing abilities [25,26,27,28,29,30].

Plants exhibit a wide variability in their response to As [31]. Hypertolerant and hyperaccumulator plants can survive on polluted soil and translocate metals or metalloids into aboveground parts of plants. Many plants have been reported to tolerate As in polluted soils, but not as hyperaccumulators [32,33]. In these hypertolerant plants without the ability of hyperaccumulation remain most of the toxic metals or metalloids in the roots and translocate just a minor of the absorbed amount [34]. Accumulated metals or metalloids such as As, which is transformed from arsenate to arsenite to reduce toxicity by arsenite reductase, is bound with a thiolic compound such as glutathion or phytochelatines and sequestrated in vacuoles [35,36]. In plants, it was observed that dual-specificity phosphatase (CDC25) may functionally mediate arsenate reductase activity involved in arsenate resistance [37,38,39,40,41,42].

Spinach (*Spinacia oleracea* L.), as one of the most valuable leafy vegetables, has been widely studied on the basis of stress responses to various metals or metalloids [43,44,45,46]. Studies [47,48,49] showed that spinach can tolerate metal or metalloid stress, due to its strong antioxidant defense system and various other physiological mechanisms. On the other hand, the sensitivity of spinach and the negative effect of As on its growth and metabolism were also observed [44,46,50,51].

Despite the response of spinach to metal or metalloid stress being reported in recent years, plant responses triggered by As in spinach, especially changes in secondary metabolite and antioxidant metabolite production as well as possible epigenetic changes by As, are not fully revealed. Therefore, the aims of this study were (1) to determine the secondary metabolite and antioxidant metabolite production in spinach grown under As stress by measuring malondialdehyde content (MDA), total phenolic content (TPC), total flavonoid content (TFC), total phenolic acids content (TPAC) and total antioxidant capacity (TAC); (2) to evaluate the change on global methylation level based on the determination of 5-methylcytosine (5-mC) levels and CDC25 expression level in different parts of spinach grown up under As stress; (3) to determine the accumulation of total As and representation of individual As species in different parts of spinach under different levels of As contamination; and (4) to detect tissue damage caused by As stress using microscopic sections. It is assumed that increasing As contamination leads to increased stress in plants and to changes in monitored parameters.

## 2. Materials and Methods

### 2.1. Plant Material

Spinach plants were grown in a greenhouse located at GPS: 50.129976, 14.373707 in partially controlled conditions (natural light conditions, air temperature 25 ± 2/20 ± 2 °C day/night, relative air humidity 65% min and 85% max), in spring 2022. Plants were grown in the garden soil substrate with nutrient content 100 mg N/L, 44 mg P/L, 124 mg K/L (AGRO CS, Rikov, Czech Republic) and pH_KCl_ 5.6. Soil was contaminated with water solutions of Na_2_HAsO_4_·7H_2_O (Alfa Aesar, Ward Hill, MA, USA) to obtain final concentrations of 5, 10 and 20 ppm of As(V) in the soil. For comparison, control samples with no added As were used. Plants were harvested 21 days after contamination in three biological replicas and stored at −80 °C. For the ICP-MS speciation analyses and total As content determination, the samples used were dried at 40 °C for 72 h to a constant weight (FD 53, Binder, Tuttlingen, Germany).

### 2.2. Analysis of Total Arsenic Content

Samples of plant material of 150 mg of dry matter per sample, together with 4 mL of HNO_3_ (Analytika, Prague, Czech Republic) and 2 mL of H_2_O_2_ (Carl Roth, Karlsruhe, Germany) were put into a 35 mL quartz vessels, capped and heated in a microwave oven (Discover SP-D, CEM Corp., Matthews, NC, USA) at 180 °C for 18 min. Milli-Q water (≥18.2 MΩ cm^−1^; MilliQ system, Millipore SAS, Molsheim, France) was used for the dilution of digested samples. Measurements were performed on a mass spectrometer with inductively coupled plasma ionization (ICP-MS; Agilent 7700x, Agilent Technologies Inc., Santa Clara, CA, USA) operating in He mode, with external calibration, and ^72^Ge as an internal standard. As a certified reference material, peach leaves were used (SRM-1547, NIST). Analyses were performed in three biological replicates with procedural blanks.

### 2.3. Arsenic Speciation Analysis

Determination of As species representation was performed on an HPLC-ICP-MS system. An anion exchange column PRP-X100 (150 × 4.6 mm, 10 µm; Hamilton, MA, USA) was used and gradient elution with 4 mmol L^−1^ NH_4_NO_3_ (A) and 60 mmol L^−1^ NH_4_NO_3_ (B) as mobile phases, both with pH adjusted to 8.7 (NH_4_OH) as in [52] and with a flow rate of 0.95 mL^−1^ min^−1^. Samples were injected in 20 µL volume to the system with a column tempered at 30 °C. Analysis starts on mobile phasis ratio 70%/30% A/B (0–3.3 min), where the gradient starts changing linearly to 20%/80% A/B (3.5–8.0 min) and returns to 70%/30% A/B. A calibration curve was created from a set of standard solutions with 0.1, 0.5, 2.5, 10 and 50 µg L^−1^ As concentrations of NaAsO_2_ (As^III^), Na_2_HAsO_4_·7H_2_O (As^V^) and dimethylarsinic acid (DMA), all from (Fluka, Buchs, Switzerland) and monomethylarsonate (MMA) synthesized in-house. A mobile phase (50%/50% A/B) was used for the dilution of samples to fit in the ICP–MS external calibration range. The ICP–MS system was internally calibrated by certified reference material ASTASOL-As (1000 ± 2 mg L^−1^) (Analytika, Prague, Czech Republic).

### 2.4. 5-Methylcytosine Levels Determination

Roots and leaves of spinach plants were crushed with a mortar and pestle in liquid nitrogen conditions. Samples of 100 mg wet weight were used for DNA isolation using a NucleoSpin Plant II isolation kit (Macherey-Nagel GmbH & Co. KG, Dueren, Germany) with a miniprep protocol that was recommended by the manufacturer using a PL1 lysis buffer. Samples of isolated DNA were used for the global DNA methylation status determination with a MethylFlash Methylated DNA Quantification Kit (Fluorometric) (Epigentek Group Inc., Farmingdale, NY, USA) by the manufacturer’s recommended protocol, and 100 ng of DNA was used for each assay. Measurement of fluorescence at 530_EX_/590_EM_ nm was set on the fluorescence microplate reader (Tecan Infinity M200, Tecan Deutschland GmbH, Crailsheim, Germany) and Magellan software for quantification.

### 2.5. Relative Transcript Level Determination

Total RNA was isolated from 100 mg of leaves and roots of spinach plants, grown in all contaminated variants and the control variant, with a NucleoSpin RNA Plant Mini Kit (Macherey-Nagel GmbH & Co. KG, Dueren, Germany) and cDNA was synthesized from 100 ng of total RNA by using a qScript cDNA Synthesis Kit (Quantabio, Beverly, MA, USA). Primers (5′-CGCTAAGCACCAGCAGTT-3′) and (5′-AGATTCGTCTTCAAGCTTAGAAGTTAATG-3′) designed by the CDC25 mRNA predicted sequence (GenBank accession no. XM_021991520.1) were used for PCR amplification of the sequence of interest with isolated RNA with using Q5 High-Fidelity DNA polymerase (New England Biolabs, Ipswich, MA, USA). The sequence was put into a pGEM-T Easy vector (Promega Corporation, Madison, WI, USA) and compared with the predicted sequence by custom DNA sequencing (Eurofins Genomics, Wien, Austria) and it was found identical to the predicted sequence. Quantitative real-time reverse-transcription PCR (qRT-PCR) gene expression analysis of CDC25 in the samples obtained from plants was performed by using iTaq Universal SYBR Green Supermix (Bio-Rad, Hercules, CA, USA) by recommended protocol. The reaction contained 0.25 μM specific primers and 1 μL of cDNA (equivalent to 100 ng of the total RNA). The concentration of RNA isolated from 100 mg of tissue using the RNeasy Plant Mini Kit was determined by measuring the absorbance at 260 nm and its integrity was checked by formaldehyde agarose gel electrophoresis. qPCR primers for CDC25 (dual-specificity phosphatase): (5′-TCGTCGACGTCCGTGATGATGAGAGA-3′) and (5′-TGGGCCCCGTACCTGACTAAGAGC-3′), were designed to overlap the intron–exon junction. The qPCR analyses on three independent samples in two technical replicates were performed on a StepOnePlus real-time PCR system (Applied Biosystems, Waltham, MA, USA). The methods used for the setting of qPCR conditions, primer designing, control of DNA absence and output data calculations were based on [53], using the 2-ΔCt1 method for the relative amount of CDC25 expression level quantification, with a control variant as reference.

### 2.6. Phenolic Compounds and Total Antioxidant Capacity

For the estimation of the content of MDA, the method of [54] was applied. Briefly, 0.5 g of fresh leaves were ground in liquid nitrogen and homogenized with 10.5 mL of 80% ethanol. The filtered extract was mixed with trichloracetic acid containing thiobarbituric acid and the mixture was heated in a waterbath at 95 °C for 25 min. This concoction was centrifuged at 10,000 rpm for 1 min after cooling and the absorption of supernatant was measured against water as a blank at wavelengths of 600, 500 and 432 nm. The gained values were used for the calculation of MDA content in nmol g^−1^ of fresh leaves according to [54]. The determination of TPC was adapted according to the method of [55]. The dried and powdered leaves of spinach (approx. 0.5 g) were mixed with 5 mL of 80% ethanol and placed in tubes to ultrasound bath Sonorex (35 kHz, Bandelin, Berlin, GE) for 15 min. An aliquot of the extract was mixed with 10-times diluted Folin-Ciocalteu’s reagent and 7% Na_2_CO_3_ was then finally added. The absorbance was measured at 715 nm against a blank (distilled water) after 90 min. TPC was calculated as milligrams of gallic acid equivalents (GAE) used for the calibration curve, per gram of dry weight. The method for measuring TFC is based on the aluminium colorimetric method used by [56]. The absorbance of the reaction mixture containing ethanolic extract from TPC analysis was measured at 415 nm. TFC was calculated as milligrams of quercetin equivalents (QE), used for the calibration curve, per gram of dry weight. The TPAC was determined according to the assay of [57] and the samples were measured at 465 nm on the same apparatus as before. TPAC was calculated as milligrams of caffeic acid equivalents (CAE) used for the calibration curve, per gram of dry weight. Determination of TAC in spinach samples was analyzed by the method described by [58]. TAC of ethanolic extracts from TPC assay was calculated as milligrams of ascorbic acid equivalents (AAE) used for the calibration curve, per gram of dry weight.

### 2.7. Microscopy

Microscopic sections were obtained by the transversal cut of a root just above the 1st root branch, towards the hypocotyl, at the most developed root and observed unstained at 100× magnification. Four variants, including the control and 5, 10 and 20 ppm of As substrate concentrations, were observed using a microscope (Nikon Eclipse 50i with Nikon DS-Fi2 camera, Nikon Corporation, Tokyo, Japan). The following parameters were observed in the microscopic sections of the observed variants—the primary cortex (development of exodermis, formation of the sclerenchyma layer) and the area of the vascular bundle of the root (the development and arrangement of xylem elements, respectively).

### 2.8. Statistical Analyses

Differences in monitored treatments defined as the rate of As induced stress were evaluated by factorial and one-way ANOVA. Significant differences between the treatments were evaluated by multiple comparisons using the Tukey HSD test after obtaining significant results (*p* < 0.05). STATISTICA Version 13 (Statsoft, Tulsa, OK, USA) software was used for analysis processing. Principal component analysis was used for the evaluation of relationships between all parameters (MDA, TPC, TFC, TPAC, TAC, 5 mC %, RTL, As) with Canoco 5 [59].

## 3. Results

### 3.1. Arsenic Contamination Impact on Phenolic Compounds and Total Antioxidant Capacity

**Figure 1:**
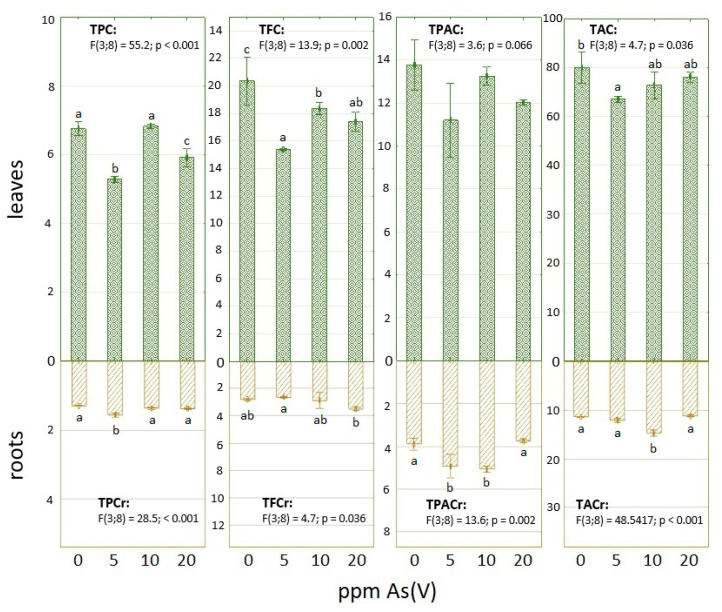
Content of phenolic compounds and total antioxidant capacity. TPC values in gallic acid equivalents (GAE) mg g^−1^ DM, TFC values in quercetin equivalents (QE) mg g^−1^ DM, PAC values in caffeic acid equivalents (CAE) mg g^−1^ DM, TAC values in ascorbic acid equivalents (AAE) mg g^−1^ DM. The letters indicate significant differences based on the post-hoc Tukey test, assuming *p* < 0.05.

Table 1 shows the content of MDA in fresh leaves and roots harvested at the end of the experiment. There is a higher content of this lipid peroxidation product in control samples of both organs than in the 5 ppm As(V) variant. The MDA concentration elevated gradually in the case of the upper part with an increased amount of used metalloid, while all MDA levels in roots were decreased in comparison with control. Despite similar concentrations of total As in leaves in the case of 5 ppm and 10 ppm As(V) variants, the MDA was measured higher in the latter samples, where the presence of As was also higher (Table 1). Generally, the contents of TPC, TFC and TPAC, which were observed in dry matter, were higher in samples prepared from leaves than roots (Figure 1). In the bottom part of spinach, TPC was rising with a higher applied concentration of arsenate; the highest increase was observed in the case of the 5 ppm As variant. On the other hand, TPC in leaves was the lowest in the case of the 5 ppm As variant and only the samples in the variant of 10 ppm arsenate in the soil had TPC comparable with control at least (Figure 1).

In the case of TFC (Figure 1), all leaves samples treated with arsenate had lower TFC than control. Regarding variants treated by arsenate, the highest value of quercetin equivalents was observed after 10 ppm As treatment again. However, the application of this metalloid slightly enhanced the content of these secondary metabolites in roots, especially in the 20 ppm As(V) variant. The measured differences of TPAC in leaves between variants were similar to TFC (Figure 1), when all samples treated by arsenate had a lower content of caffeic acid equivalents than control. All spinach plants also had a lower shoot/root ratio for these metabolites in comparison with the flavonoids, where the TFC was on behalf of leaves more (Figure 1). Contrary to flavonoids, TPAC was increased in samples, with arsenate concentrations of 5 and 10 ppm in soil, in comparison with control (Figure 1). The total antioxidant capacity of prepared extracts is expressed as ascorbic acid equivalents (Figure 1). This acid also belongs to a group of antioxidants and is also part of various pathways for the regeneration of other protective molecules, such as GSH. Differences in TAC values in leaves are similar to the MDA results (Table 1). The highest amount of AAE was observed in the control sample, while the plants in 5 ppm of arsenate contamination had the lowest TAC. This parameter was then increased with a higher concentration of this metalloid. The values of TAC in roots were also rather increased after arsenate application; only 20 ppm As contamination did not take effect in comparison with control (Figure 1).

### 3.2. Arsenic Accumulation and Speciation in As(V) Exposed Spinach Plants

**Figure 2:**
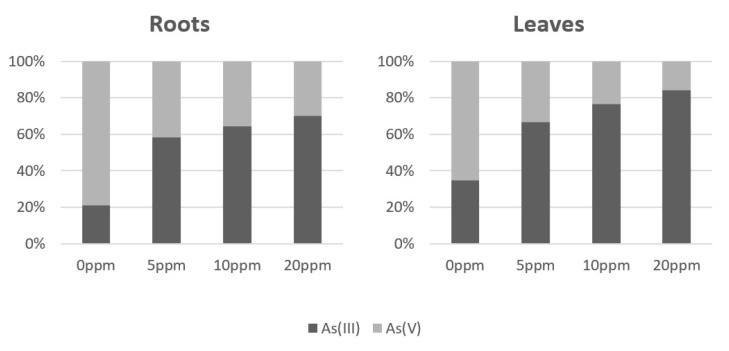
Arsenic species ratio (%).

Arsenic accumulation (Table 1) was unevenly distributed between roots and leaves. In the roots, most of the accumulated arsenic in all the As(V) contaminated variants was deposed; only units of percentage accounted for As transported from roots to leaves. Arsenic species representation (Figure 2) was strongly in favor of the As(III) specie in the all As(V) contaminated variants; a share of As(III) rose along with the amount of added As(V). That suggests enzymatic catalyzed arsenate reduction and growing activity with increasing As contamination.

### 3.3. Levels of 5-Methylcytosine under Arsenic Stress

**Figure 3:**
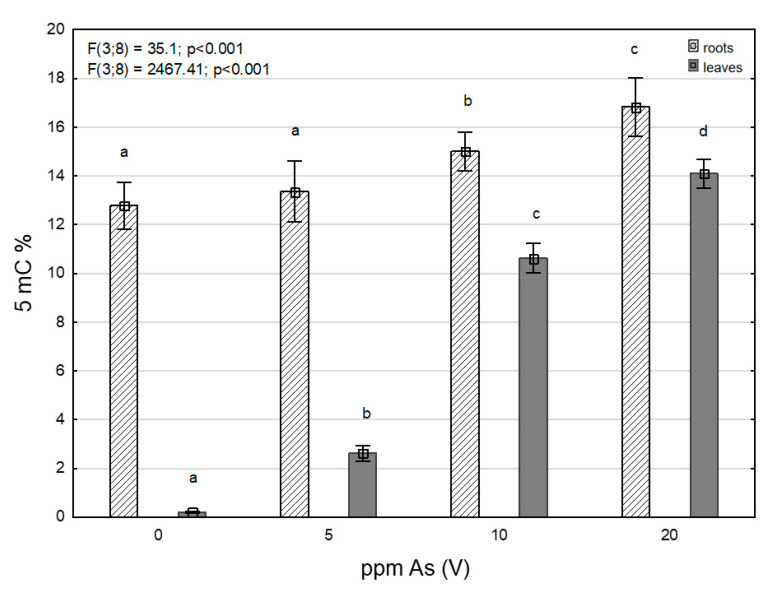
Results of the factorial ANOVA analysis for levels of 5-methylcytosine. The error bars indicate the standard deviations and the bar height is the mean value. Categories on the X-axis show four treatments (control, 5 ppm, 10 ppm and 20 ppm) values in roots and leaves. The letters indicate significant differences based on the post-hoc Tukey test, assuming *p* < 0.05.

Levels of 5-mC (Figure 3) in roots and leaves showed an increasing trend in all contaminated variants, together with growing As contamination (Table 1), pointing to As-induced DNA hypermethylation. Growth of 5-methylcytosine levels in leaves was quite significant compared to roots where, on the contrary, it was rather gradual. This was different from the content of total As, where the increase was more pronounced in the roots. However, levels of 5-mC were higher in roots than in leaves when compared in each individual contamination variant.

### 3.4. Anatomic Changes in Tissues of Plants Exposed of Arsenic

**Figure 4:**
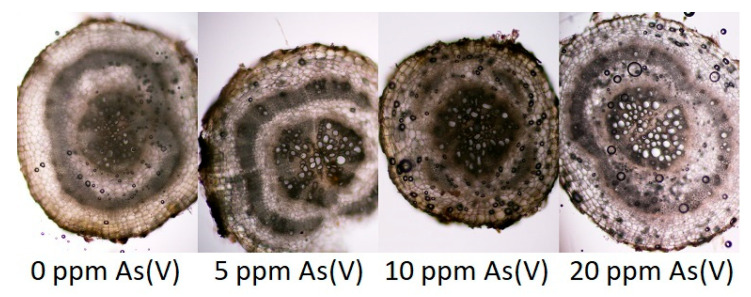
Differences in the arrangement of the rhizodermis with a primary cortex, endodermis and vascular bundle with (100×) magnification.

Contamination of the substrate with As manifested itself in differences in some monitored anatomical structures (Figure 4), especially in the development of the exodermis, in the area of the vascular bundle of the root (secondary xylem) and further in the formation of the mechanical tissue (sclerenchyma). From all variants, 5 ppm and 10 ppm reacted more sensitively than others. In them, the function of the exodermis (thus, also the apoplastic barrier) was apparently disturbed, incipient necrosis appeared locally in the parenchyma of the primary cortex and the development of the secondary xylem was also disturbed. Changes were also manifested in the development of the mechanical tissue (sclerenchymatic cylinder of the primary cortex). The 20 ppm variant differed minimally from the control variant only in the greater lightness of the conductive elements of the secondary xylem, which may be related to the level of transport of aqueous solutions from the substrate.

### 3.5. Arsenic-Dependent CDC25 Transcription

**Figure 5:**
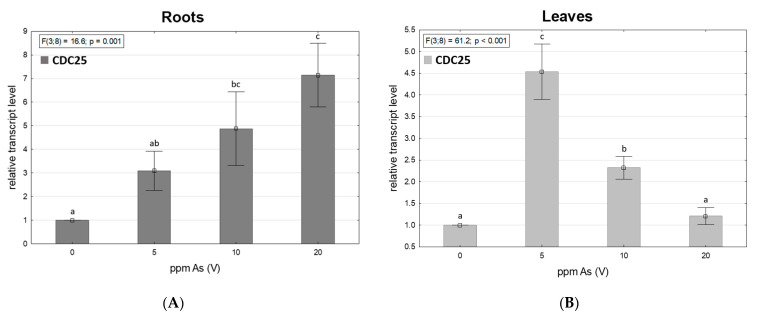
Expression of CDC25 gene in spinach roots: (**A**); and leaves: (**B**) measured by qRT-PCR from 3 plants grown in all experimental variants. Values are mRNA average relative levels ± standard deviation of means in three biological replicates. The letters indicate significant differences based on the post-hoc Tukey test, assuming *p* < 0.05.

Figure 5 shows the relative transcript level of CDC25 changes in roots and leaves of spinach. In roots of spinach, a growing trend was observed in the expression of the CDC25 gene with growing As contamination. In leaves, the expression rate was highest in the 5 ppm As(V) contaminated variant, followed by a sharp decline of CDC25 expression in 10 and 20 ppm As(V) variants, even though the CDC25 transcription rate was, in all contaminated variants, higher than in control variants. Results indicate the role of CDC25 in plant As defense, which could be a useful insight for further research such as functional complementation assays.

## 4. Discussion

In the compared studies [60,61], which focused on crops grown in soil contaminated with toxic elements, similarities were found with the results of this study. The content of As in the edible parts of spinach from the control variant grown in soil without added As contamination was below the hygienic limit for As (1 µg g^−1^). Values of As content from our samples fit into the range of values (3.4 µg g^−1^ to 78.0 µg g^−1^) [62,63] from spinach grown in As-contaminated soil.

It was observed [64] that spinach had the highest shoot concentration of As in comparison with other leafy vegetables (*Lactuca sativa* L., *Chrysanthemum coronarium* L.) during hydroponical cultivation. The concentration of As was higher with an increasing amount of applied metalloid and the roots also contained more of this element than shoots. Used Na_2_HAsO_4_ (400 μM) increased MDA levels in both types of organs; this compound was also present in control. For this concentration, they described the elevated activity of enzymes such as superoxide dismutase, catalase or peroxidase in shoots, while their activity was not significant or it was decreased against control in roots. They also tested and determined the safety threshold for the As soil amount for spinach cultivation to a value of 76.2 ppm.

A study with the same As compound [65] was in the form of heptahydrate and one more solution (600 μM) in addition. The experiment on *S. oleracea* var. Jyoti green took place in pots and the results were evaluated after 60 days. MDA was only observed in the leaves and the significant increase of lipid peroxidation was measured in the samples, where 600 μM solution was applied. Similar results were found in our study, where lower amounts of As did not take such effect on MDA levels in comparison with control (Table 1). Besides the morphological changes, these authors also described a decreased number of vascular bundles in the leaves and other negative anatomical changes as a consequence of As stress.

An increased content of lipid peroxidation product was also observed in the study of [43] in shoots and roots after the application of lower concentrations of Na_2_HAsO_4_·7H_2_O (50 and 100 μM). Besides the activity of enzymatic antioxidants, they determined the amount of non-enzymatic protectants against oxidative stress, such as phenolics, flavonoids and others. For the roots, the results were alike, when samples with a lower intensity of As stress had a higher TPC and TFC. On the other side, these compounds did not show similar responses in our survey. Antioxidant enzymes had analogous results as secondary metabolites in both organs. There was a stronger response of antioxidant enzymes after the application of sodium arsenate (60 μM), but a weaker one in the case of a more concentrated solution (120 μM) was also described by [46].

**Figure 6:**
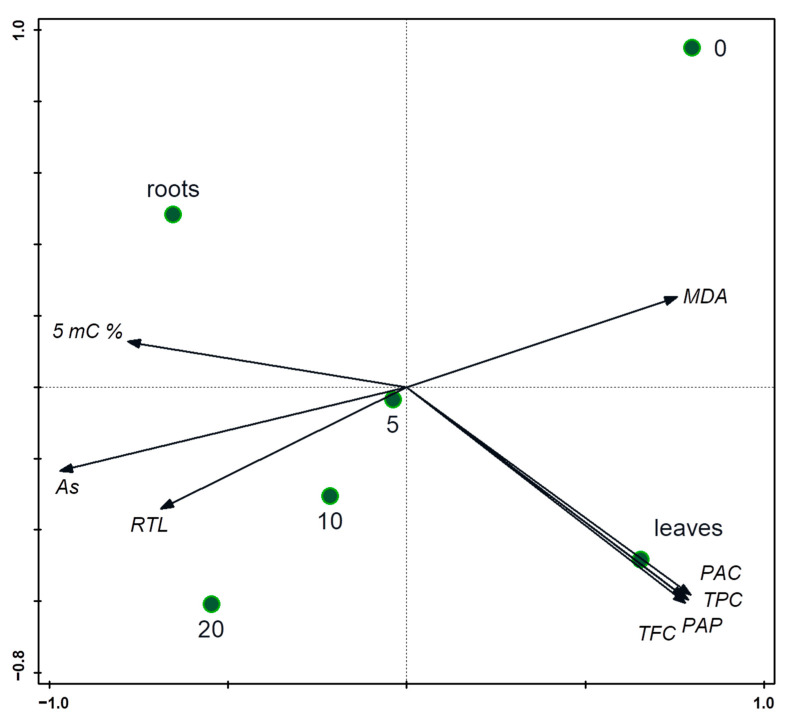
Relationships between the treatments and measured properties of the spinach evaluated using PCA (principal component analysis) with supplementary variables. The treatments are displayed using color circles and the arrows are measured variables or properties of the plants. The first two axes explain 91.45% of the total variation in the plant’s properties. RTL: relative transcript level.

Regarding phenolic compounds in general, more represented in leaves (Figure 6), [66] observed TPC and TFC in dried leaves after the application of As_2_O_3_ in four increasing concentrations (25, 50, 75, 100 mM). The content of the analyzed group of metabolites, which were expressed in gallic acid and quercetin equivalents as well, was the highest in the case of control. TPC and TFC, in particular in As-treated samples, were decreasing, but there were various fluctuations. The lower content of flavonoid that the authors discussed with respect to the findings of [67], as a result of As(III) binding to various enzymes, was involved in respective biosynthetic pathways. Figure 2 shows this form of the metalloid is dominant in leaves and increases with higher used concentration. The decreased levels of flavonoids also described in [68], but where a low concentration of arsenate was applied, took a positive effect on the amount of these metabolites in the tomato leaves.

Flavonoids can participate on heavy metal protection [69], when the mutants of *Arabidopsis thaliana* L. with altered flavonoid pathway had worse growth under cadmium treatment than the control plant. The addition of metabolites such as quercetin or naringenin then improved the observed characteristic.

The production of MDA under heavy stress can also be dependent on the type of used compound [70]. Some metals, such as Co or Ni, enhanced lipid peroxidation, while the activity of antioxidant enzymes was not sufficient in spinach leaves. The negative effect of elements (Cu, Cd, Pb) also manifested in wheat roots [71]. However, the phenolic acids content was increased, which was discussed with regard to their engagement with the lignification process in roots as a defense against stress compounds and the As could have a similar effect on spinach’s roots (Figure 1). Nevertheless, phenolic compounds, such as ferulic acid, are not only non-enzymatic antioxidants in spinach, but also, the content of these molecules participating in antioxidant activity in leaves can be decreased by metals [72].

By observing sections of spinach root under a microscope from plants grown in an As(V)-contaminated environment, it was found that 5 ppm and 10 ppm variants reacted more sensitively than the control variant and 20 ppm variant. Tissues in spinach roots from 5 ppm and 10 ppm contaminated variants were visibly more damaged than roots from the control variant and 20 ppm variant. Tissue damage could have been caused by the toxic action of the As(III), which was found in roots as a major specie. In roots of vegetables grown in an As(III)-contaminated environment, it was found [73,74] that the highest contamination of As(III) may not affect the highest cytotoxic response and may not affect the highest tissue damage.

Stress induced by toxic metals can affect plants on epigenetic levels and cause different reactions such as the demethylation or hypermethylation of DNA [75,76]. DNA hypermethylation is mostly connected with decreased gene expression [26]. The growth of 5-mC levels in leaves was significantly steeper and a sharp increase of methylated DNA levels in 10 and 20 ppm As-contaminated variants was probably the reason for lower CDC25 expression levels in leaves. Levels of 5-mC in roots were higher than in leaves, but the increase was more gradual and the increase in CDC25 expression was not affected; and with increasing As contamination, the level of CDC25 expression also increased, as did the (As III) ratio. Arsenate reduction in plants by CDC25 was described in [37,38]. The growth of the As (III) ratio was in leaves too, but the values of accumulated As were much lower than in the roots and the reduction of arsenate by CDC25 may have worked as the activity of the enzyme may have been sufficient despite the lower expression.

## 5. Conclusions

A decreasing trend of MDA content with growing As contamination in roots of spinach was significant. In addition, the amount of accumulated arsenic was mostly deposed in the roots, unlike leaves where the accumulation of As was significantly lower. In leaves, a sharp increase of the 5-mC level was observed, with growing As contamination, but 5-mC levels were higher in roots when comparing individual variants. Leaves contained multiple times higher values of all measured phenolic compounds than roots. Arsenic As(V) and As(III) species ratios representation, in plants grown on soil contaminated with different amounts of As(V), were in favor of As(III) and ratio growing with arsenate concentration addition, pointing to an arsenate reductase-like enzymatic activity. CDC25 transcript level grown in roots with growing added amounts of As, in leaves, was highest in the lowest contaminated variant. The toxic effects of As were clearly visible in tissue changes in the roots of experimental plants. The conclusion of the experiment found a noticeable impact on metabolism, caused by As contamination, but a low accumulation of As in edible parts of spinach (leaves).

## Figures and Tables

**Table 1:** Arsenic and MDA content in different parts and variants (As µg g^−1^ DM), (MDA nmol g^−1^).

ppm As(V)	Total Arsenic Content		MDA	
	Roots	Leaves	Roots	Leaves
Control	2.58 ± 0.26 ^a^	0.31 ± 0.06 ^b^	11.97 ± 0.54 ^d^	10.84 ± 0.16 ^a^
5	72.99 ± 3.03 ^b^	5.23 ± 0.30 ^a^	8.70 ± 0.82 ^c^	7.87 ± 1.01 ^b^
10	180.51 ± 5.06 ^c^	5.16 ± 0.09 ^a^	4.72 ± 0.48 ^b^	10.24 ± 1.04 ^a^
20	302.69 ± 11.83 ^d^	23.39 ± 2.39 ^c^	2.35 ± 0.43 ^a^	11.60 ± 0.62 ^a^
*p*	<0.001	<0.001	<0.001	0.002

The letters indicate significant differences based on the post-hoc Tukey test (*p* < 0.05).

## Data Availability

The data presented in this study are available in this article.
